# Supplementary material for: Genetic Interference of FGFR3 Impedes Invasion of Upper Tract Urothelial Carcinoma Cells by Alleviating RAS/MAPK Signal Activity
Source: Int J Mol Sci. 2023 Jan 16;24(2):1776. doi: 10.3390/ijms24021776 (PMC9863353; doi:10.3390/ijms24021776)
Supplement: Supplementary file 1 [file ijms-24-01776-s001.zip › ijms-2037532-supplementary.pdf]

Supplementary Figure S1

A

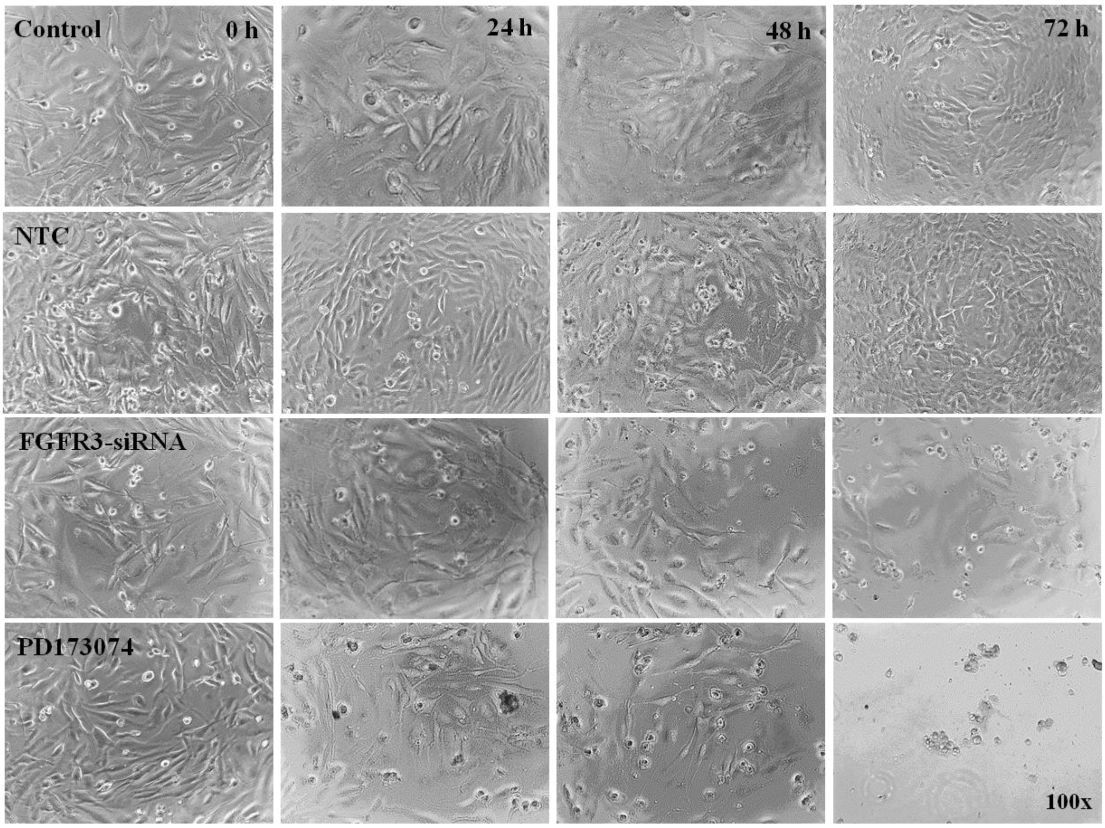

B

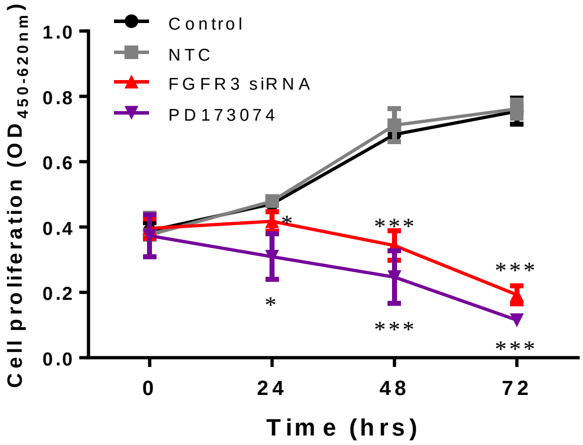

Figure S1. Morphology and proliferation of BFTC-909 UTUC cells treated with FGFR3 siRNA or FGFR kinase inhibitor. The cells were treated with either FGFR3-targeted siRNA, scramble non-target control (NTC), or PD173074 at 10 nM for consecutive 72 h. (A) The cell morphology was documented under phase contrast microscope at 24 h intervals. Original magnification: 100× (B) Alternatively, the control and treated cells were subjected to WST-1 proliferation assay. Optical density (OD) data are expressed as mean±SEM (n=3). * *P*<0.05, *** *P*<0.001 vs. respective control groups.
